# Enhancement of the Replication of Hepatitis C Virus Replicons of Genotypes 1 to 4 by Manipulation of CpG and UpA Dinucleotide Frequencies and Use of Cell Lines Expressing SECL14L2 for Antiviral Resistance Testing

**DOI:** 10.1128/AAC.02932-15

**Published:** 2016-04-22

**Authors:** Jeroen Witteveldt, Marion Martin-Gans, Peter Simmonds

**Affiliations:** a Roslin Institute, University of Edinburgh, Easter Bush, Edinburgh, United Kingdom; b Nuffield Department of Medicine, University of Oxford, Oxford, United Kingdom

## Abstract

Treatment for hepatitis C virus (HCV) has improved greatly through the use of direct-acting antivirals (DAAs). However, their effectiveness and potential for drug resistance development in non-genotype 1 variants of HCV remain relatively unexplored, as *in vitro* assays to assess drug susceptibility are poorly developed and unsuited for a transient-transfection format. In the current study, we have evaluated the effects of dinucleotide frequency changes in the replicon and the use of a SEC14L2-expressing cell line on the replication of HCVs of different genotypes and evaluated the resulting assay formats for measurements of susceptibility to the DAA sofosbuvir. Removal of CpG and UpA dinucleotides from the luciferase gene used in HCV replicons of genotype 1b (Con1) and genotype 2a (JFH-1) achieved between 10- and 100-fold enhancement of replication over that of the wild type posttransfection. Removal of CpG and UpA dinucleotides in the neomycin gene or deletion of the whole gene in replicons of genotype 3a (S52) and genotype 4a (ED43) enhanced replication, but phenotypic effects on altering luciferase gene composition were minimal. A further 10-fold replication enhancement of replicons from all four genotypes was achieved by using a transgenic Huh7.5 cell line expressing SECL14L2, whose expression showed a dose-dependent effect on HCV replication that was reversible by small interfering RNA (siRNA) knockdown of gene expression. By combining these strategies, the 100- to 1,000-fold enhancement of replication allowed the susceptibility of all four genotypes to the RNA polymerase inhibitor sofosbuvir to be robustly determined in a transient-transfection assay format. These methods of replication enhancement provide new tools for monitoring the susceptibility and resistance of a wide range of HCV genotypes to DAAs.

## INTRODUCTION

Hepatitis C virus (HCV) is a positive-sense RNA virus of the Flaviviridae family first described in 1989 (1) and subsequently identified as the principal cause of non-A, non-B hepatitis in blood recipients and hemophiliacs as well as widely infecting injection drug users (2, 3). Based on a meta-analysis of serological data, it is estimated that ∼185 million people worldwide are infected with HCV (4). Currently, there are 7 genotypes of HCV recognized, of which genotype 1 accounts for approximately 45% of all infections, followed by genotype 3, accounting for 30%, and genotypes 2, 4, and 6, accounting for the remaining 25% (5). It is estimated that 70 to 80% of all new infections progress to chronicity that can ultimately lead to end-stage liver disease (6, 7).

Until recently, HCV was treated by combination therapy of pegylated interferon alpha and the nucleoside inhibitor ribavirin, which is highly effective in genotype 2 and 3 infections (∼80% of cases show a sustained virological response) but less so in genotype 1 and 4 infection, where 50% or fewer cases achieve virus clearance (8–10). The phenotypic diversity of HCV genotypes, as manifested by these major differences in treatment responses, therefore requires the effectiveness of and the development of resistance to novel antiviral treatments to be evaluated separately for different HCV genotypes and subtypes. Such testing is generally performed by using subgenomic replicons in which the structural genes are replaced by a luciferase or other reporter gene to allow replication to be quantified (11, 12). However, most wild-type (WT) replicons replicate poorly in cell culture and are generally restricted to the Huh7.5 cell line, in which a number of cellular defense pathways are nonfunctional, including the cytosolic RNA receptor RIG-I (13). However, under antibiotic selection for replicon-containing cells, stable cell lines in which replicons rapidly acquire cell culture-adaptive mutations that enhance replication can be selected. For example, in the genotype 1b Con1 replicon, adaptive mutations occur in the NS3, NS4B, and NS5A genes (12, 14, 15), which may enhance HCV protein-protein interactions and viral morphogenesis, although the mechanisms remain poorly understood (16). Such mutants, however, show increases in viral RNA levels in transient-replication assays without antibiotic selection. Although there is now a wide range of stably transfected Huh7.5 cell lines containing replicons of different HCV genotypes, the existence of functionally poorly defined adaptive mutations has the potential to influence their susceptibility to direct-acting antivirals (DAAs) and the effects of DAA-associated mutations on antiviral susceptibility. While transient-replication assays are clearly preferable for such testing, many of the currently available replicons do not have the required level of replication needed in this assay format to accurately estimate changes in replication levels in drug inhibition studies.

In this study, we have investigated the effectiveness of two novel approaches to enhance HCV replication in cell culture. The first approach is to reduce the frequencies of CpG and UpA dinucleotides in the reporter genes of HCV replicons to enhance replication. The rationale is based on previous studies that demonstrated that lowering CpG and UpA frequencies in coding regions of echovirus 7 or the luciferase gene in a derived replicon substantially enhanced their replication over that of the wild-type virus (17, 18). A second, separate approach is based upon the recent findings that expression of the SEC14L2 gene is a limiting factor in HCV cell culture replication and that enhancement of HCV replication can be achieved through the use of cell lines that overexpressed the SEC14L2 gene, increasing the number of CFU in antibiotic-selected non-transient-replicon studies (19).

## MATERIALS AND METHODS

### Replicon construction and RNA *in vitro* transcription and translation.

**FIG 1:**
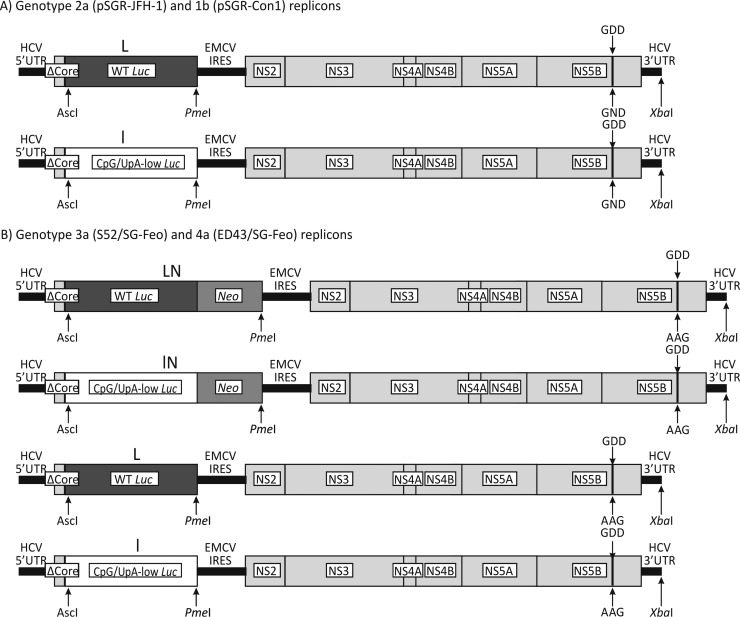
Structures of subgenomic replicons and mutants. Shown are diagrammatic representations of the replicons used in this study, with changes to the luciferase gene (L and l), deletion of the neomycin gene (N), and replacement of the GDD motif in NS5B with GND (or AAG in genotypes 3 and 4) to make it replication defective labeled.

The Con1 subgenomic replicon was provided by R. Bartenschlager (11), SGR-JFH1 and SGR-JFH1/GND were provided by J. McLauchlan (20), and S52/SG-Feo(AII) and ED43/SG-Feo(VYG) were obtained from C. Rice (21). A synthetic DNA sequence used to replace the WT luciferase sequence was specified by using the program Sequence Mutate in the SSE package (22) and was cloned in the various replicons by using unique restriction sites at the 5′ end (AscI) and the 3′ end (PmeI) of the luciferase coding sequence (Fig. 1).

Replicon plasmids were linearized with XbaI, treated with mung bean nuclease, purified, quantified, and used as the template for *in vitro* RNA transcription (MEGAscript; Invitrogen). RNA was precipitated by using LiCl, washed, resuspended in water, aliquoted, and stored at −80°C.

Transcript RNAs (125 ng) were used in nuclease-treated rabbit reticulocyte lysate translation assays (Promega) to compare the translation speeds of the different luciferase constructs according to the manufacturer's instructions. Samples were taken after 30 min of incubation, and luciferase expression levels were quantified as described below.

### Cell lines, electroporation, and luciferase quantification.

Huh7.5 cells were maintained in Dulbecco's modified Eagle's medium (DMEM; Invitrogen) supplemented with 4,500 mg/liter glucose, 2 mM l-glutamine, 10% heat-inactivated fetal calf serum (FCS; Harlan Sera-Lab), nonessential amino acids, 20 mM HEPES, 100 U/ml penicillin, and 100 μg/ml streptomycin and incubated at 37°C in 5% CO_2_ with 100% relative humidity.

For electroporation, cells were washed three times in cold phosphate-buffered saline (PBS), counted, and diluted to 10^7^ cells/ml. Using 4-mm electroporation cuvettes, 400 μl of the cell suspension was mixed with 1 μg of replicon RNA and electroporated (at 270 V and 950 μF) using the exponential setting in an electroporator (Genepulser XCell; Bio-Rad). Cells were immediately resuspended in warm, complete DMEM and transferred to the appropriate-sized cell culture dishes. At the desired time points, medium was removed, the cells were washed with PBS and lysed in passive lysis buffer (Promega), and luciferase expression was measured by using the Steady-glo assay system (Promega) and a luminometer (Glomax Multi detection system; Promega).

To obtain stable cell lines expressing SEC14L2, a lentiviral vector expressing this gene (Applied Biological Materials) was used to generate lentiviruses according to the manufacturer's instructions. Huh7.5 cells were transduced with the lentivirus particles and selected with 5 μg/ml of puromycin. Single colonies were isolated and grown for further evaluation.

### Protein isolation and Western blotting.

Cell monolayers were washed with PBS before being lysed in radioimmunoprecipitation assay (RIPA) buffer (50 mM Tris, 150 mM NaCl, 0.1% SDS, 0.5% sodium deoxycholate, and 1% Triton X-100), mixed with sample buffer (Laemmli 2× concentrate; Sigma), and boiled for 10 min. Protein samples were loaded onto 10% gels (Mini-Protean TGX; Bio-Rad), transferred by semidry blotting onto Immobilon paper (Millipore), and blocked in 5% milk powder in PBS. After washing in PBS-T (PBS with 0.05% Tween), blots were probed with anti-SEC14L2 (Santa Cruz Biotechnology) or anti-β-tubulin (Abcam) as a loading control for 1 h at room temperature; washed; probed with anti-mouse horseradish peroxidase (HRP) or anti-rabbit HRP, respectively, for 1 h at room temperature; washed; and developed by using ECL (ECL Prime; Amersham).

### siRNA transfection.

Knockdown of SEC14L2 was performed by using the commercially available and validated small interfering RNA (siRNA) EHU146781 (Sigma-Aldrich). The irrelevant siRNA sequences used as a control were obtained from the same manufacturer (EHUEGFP). To knock down SEC14L2 expression in the stable cell line, a titration of SEC14L2-specfic siRNA was performed by transfecting 0, 100, 200, and 400 ng of siRNA/24-well plate by using the Lipofectamine RNAiMAX transfection reagent (Invitrogen) according to the manufacturer's instructions. Mock-treated cells received 400 ng of validated nontargeted control siRNA. After 48 h, SEC14L2 expression levels were quantified by Western blot analysis and ImageJ software.

### Sofosbuvir susceptibility testing.

For the titration of sofosbuvir (PSI-7977; Cayman Chemical), cells were electroporated with replicon RNA and seeded at the required density. After 4 h, the medium was replaced with DMEM containing the desired concentration of sofosbuvir dissolved in ethanol or the mock treatment control (ethanol) and refreshed every 24 h to ensure a constant presence of sofosbuvir in the medium.

### qRT-PCR.

SEC14L2 mRNA expression levels were quantified by quantitative reverse transcription-PCR (qRT-PCR) using the forward primer 5′-TGCAGTGATCCTGGCATCTATG-3′ and the reverse primer 5′-TGAGGCTTTGTCTGGAAGCAG-3′. RNA was extracted by using the RNeasy kit (Qiagen), treated with DNase, and reverse transcribed by using GoScript reverse transcriptase (Promega). qRT-PCR was performed in a Rotorgene real-time PCR cycler (Qiagen) using the SensiFAST Sybr kit (Bioline) and glyceraldehyde-3-phosphate dehydrogenase (GAPDH) for normalization (forward primer 5′-GAAATCCCATCACCATCTTCCAGG-3′ and reverse primer 5′-GAGCCCCAGCCTTCTCCATG-3′). The RNA stability of the different replicons was determined by measuring luciferase RNA levels at 4 h postelectroporation (p.e.) by quantitative PCR (qPCR) (forward primer 5′-CCCTGGTTCCTGGAACAATTGC-3′ and reverse primer 5′-AAGAATTGAAGAGAGTTTTCACTGC-3′).

## RESULTS

### Effect of CpG/UpA dinucleotide composition on replication of HCV replicons.

In its unmodified state, the genotype 2a replicon (JFH1) already shows a robust level of replication in a number of cell lines, without any need for cell culture-adaptive mutations (20, 23, 24). We used the JFH1 replicon expressing the firefly luciferase gene driven by the HCV 5′ untranslated region (UTR) (20). The WT luciferase sequence shows a striking elevation of the ratio of the observed frequency to the expected frequency of CpG dinucleotides (1.21) (Table 1) compared to that in HCV (0.71 to 0.74 for genotypes 1 to 6) and in human mRNA sequences (mean, 0.43). High frequencies of this dinucleotide and UpA substantially restricted the replication of echovirus 7 (17), and we reasoned that this may also influence the replication capability of the HCV replicons in mammalian cell culture. We therefore replaced the wild-type luciferase gene (L) in JFH1 with the CpG/UpA-low mutant (l) in both the replication-competent (containing the enzymatically active amino acid motif GDD) and replication-incompetent (GND [genotypes 1 and 2] or AAG [genotypes 3 and 4]) backgrounds. The modified luciferase sequence was mutated to remove all 100 of the CpG dinucleotides present in the native sequence and 64 of the 86 UpA dinucleotides, the maximum possible while retaining amino acid coding identical to that of the WT sequence (Table 1 and Fig. 1A). The modified sequence, however, had G+C content, codon adaptation index (CAI), and codon pair score (CPS) similar to those of the WT sequence.

**TABLE 1:** Composition and coding parameters of WT and CpG/UpA-low luciferase sequences

Sequence	Symbol	No. of substitutions^*a*^	% C+G content	No. of CpG dinucleotides	ΔCpG^*b*^	Ratio of O/E frequencies for CpG^*c*^	No. of UpA dinucleotides	ΔUpA^*b*^	Ratio of O/E frequencies for UpA	CAI^*d*^	CPS^*e*^
WT	L		0.44	100		1.21	86		0.70	0.719	−0.011
CpG/UpA-low	l	174	0.45	0	100	0	22	64	0.17	0.804	0.021

a Number of sequence changes from the WT sequence.

b Changes in the numbers of CpG and UpA dinucleotides.

c Ratio of observed to expected frequencies of CpG and UpA dinucleotides.

d Calculated by using the CAIcal website (http://genomes.urv.es/CAIcal/) (40).

e Codon pair score; calculated as previously described (26, 41).

**FIG 2:**
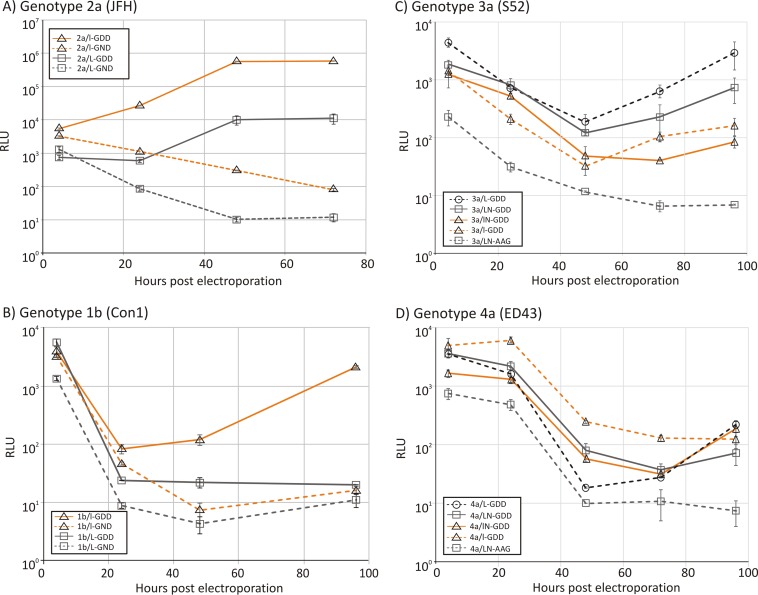
Replication of unmodified replicons and mutants with low-CpG/UpA luciferase. Shown are data for the replication of genotype 2a (A), 1b (B), 3a (C), and 4a (D) replicons after electroporation into Huh7.5 cells. Luciferase activity was measured at four time points (*x* axis) and plotted as absolute values (*y* axis). Error bars represent standard deviations. RLU, relative light units.

Genotype 2a replicons with WT and CpG/UpA-low luciferase reporter genes (replicons 2a/l-GDD and 2a/L-GDD, respectively) were transfected into Huh7.5 cells, and luciferase expression was quantified at different time points postelectroporation (Fig. 2A). To ensure that equal amounts of RNA were delivered in the cells, an aliquot of cells was taken 15 min after electroporation and treated with RNase to remove extracellular RNA, and the internalized luciferase RNA was quantified by qRT-PCR. No significant differences in the amounts of RNA were found between the different mutants used (data not shown). Despite this equal delivery of RNA, the replication kinetics of 2a/L-GDD (original) and 2a/l-GDD (CpG/UpA minimized) replicons were distinct, with approximately a hundredfold (2-log) difference in luciferase expression levels at 48 h and 96 h. This difference was greater than the 5-fold difference in luciferase gene expression levels at 4 h posttransfection and at earlier time points before significant replication of HCV had taken place (see Fig. S1 in the supplemental material).

The same changes were also made in the genotype 1b background, using the Con1 subgenomic replicon (1b/L-GDD, 1b/l-GDD, and the replication-defective counterparts 1b/L-GND and 1b/l-GND) (Fig. 2B). Unlike the genotype 2a replicons, the WT (1b/L-GDD) replicon showed very poor replication levels in Huh7.5 cells (Fig. 2B) in the first 24 h. RT-qPCR analysis confirmed that comparable amounts of RNA were electroporated in the cells at 1 h (data not shown). Similarly, from 24 h, their replication kinetics were distinct, where the CpG/UpA-low luciferase replicon showed >2-log-higher luciferase levels at 96 h postelectroporation than those of 1b/L-GDD.

To investigate the effects of dinucleotide composition changes for other genotypes, a range of replicons were constructed by using genotype 3a [S52/SG-Feo(AII)] and genotype 4a [ED43/SG-Feo(VYG)] backbones (21). The original replicons expressed luciferase as a fusion with neomycin (N), which also possesses high frequencies of CpG and UpA dinucleotides (Fig. 1B). Mutants were constructed in which luciferase was replaced with the CpG/UpA-low sequence (3a/lN-GDD and 4a/lN-GDD), those with a deletion of the neomycin gene (3a/L-GDD and 3a/L-GND), and a combination of CpG/UpA-low luciferase sequences and a neomycin deletion (3a/l-GDD and 4a/l-GDD).

Both of the original replicons, 3a/LN-GDD and 4a/LN-GDD, of genotypes 3 and 4 replicated poorly in transient-replication assays compared to genotype 2a (Fig. 1C and D). In contrast to previous experiments using genotype 1b- and 2a-based replicons, replacement of the luciferase sequence in genotype 3 (3a/lN-GDD) did not enhance replication. However, deletion of the neomycin sequence in 3a/L-GDD increased replication compared to that of 3a/LN-GDD, although this effect was not reproduced in the low-luciferase version of the neomycin-deleted construct (3a/l-GDD). For genotype 4, both the deletion of the neomycin gene (4a/L-GDD) and the introduction of CpG/UpA-low luciferase (4a/lN-GDD) enhanced replication over that of the original replicon (4a/LN-GDD), effects that were synergistic at early time points (4a/l-GDD). For all genotype 3 and 4 replicon mutants, quantitation of transfected RNA at 1 h demonstrated that comparable amounts of RNA were electroporated into the cells (data not shown).

### Effect of SEC14L2 expression on HCV replication.

**FIG 3:**
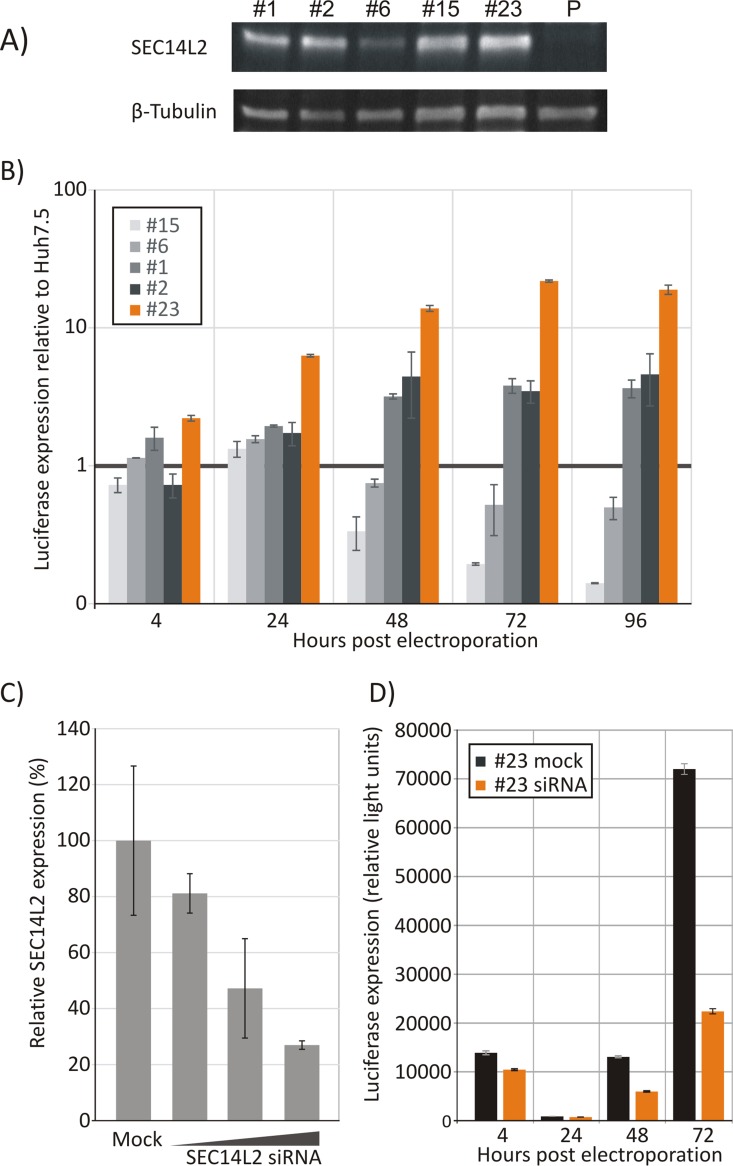
Expression of SEC14L2 in different cell lines and effect of HCV replication. (A) Quantification of SEC14L2 expression by Western blotting of cell lysates from different cell lines. Samples were normalized to the total amount of protein loaded; a control immunoblot using β-tubulin is shown at the bottom. P, parental Huh7.5 cell line. (B) Luciferase expression in different cell lines at different time points after electroporation of 1b/l-GDD. The values are normalized to luciferase expression of the 1b/l-GDD replicon in the parental Huh7.5 cell line at each time point. (C) Effect of transfection of SEC14L2-specific siRNA in cell line 23 on SEC14L2 expression levels as determined by Western blotting. (D) Replication of 1b/l-GDD in siRNA-treated and irrelevant siRNA-treated 23 cells. Error bars depict standard deviations.

To investigate whether the reported enhancement of replication by the expression of SEC14L2 (19) could also be achieved in a transient-transfection replication assay, a number of clonal cell lines stably expressing SEC14L2 were made. Based on SEC14L2 expression levels measured by RT-qPCR, several lines were selected and tested for SEC14L2 protein expression levels by Western blotting (Fig. 3A). All five cell lines selected constitutively expressed detectable but variable levels of SEC14L2 protein, while it was undetectable in the parental Huh7.5 cell line (labeled “P”).

Three out of five cell lines supported enhanced replication of the 1b/L-GDD replicon (Fig. 3B), with cell line 23 showing the highest increase in replication compared to the parental cell line (∼25-fold). To verify that this replication enhancement arose directly from an increased expression of SEC14L2 and was not an artifact introduced by transduction and consequent single-cell selection, siRNA was transfected into 23 cells to knock down SEC14L2 protein expression (Fig. 3C). The effect of the SEC14L2 knockdown on HCV replication was investigated in cells transfected with a siRNA concentration that achieved a 75% knockdown of expression (Fig. 3D). These cells were electroporated with 1b/l-GDD, and luciferase expression was measured and compared to that in mock-treated 23 cells (Fig. 3D). Knockdown of SEC14L2 decreased replicon replication by ∼4-fold at 72 h, confirming the involvement of SEC14L2 in the control of replicon replication.

### Replication of HCV genotypes 1 to 4 in SEC14L2-expressing cells.

**FIG 4:**
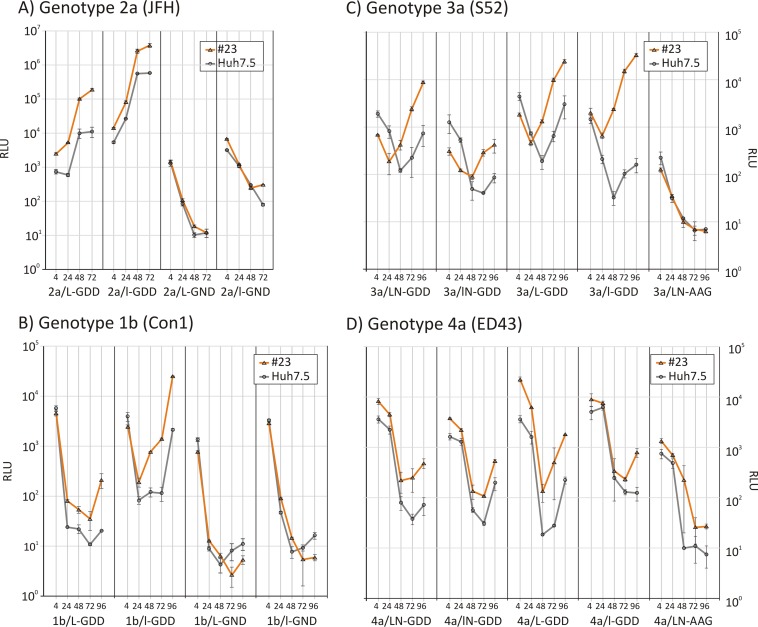
Comparison of replication of replicons from genotypes 1 to 4 in the Huh7.5 and 23 cell lines. Shown are data for the replication of replicons from genotypes 1 to 4 generated in this study in the Huh7.5 and 23 cell lines; the right panels show luciferase expression from the replication-incompetent control. Error bars depict standard deviations.

Replicons with all four genotype backbones and the corresponding CpG/UpA-low luciferase mutants were electroporated into the SEC14L2-expressing cell line 23, and replication was compared to that in parental Huh7.5 cells (Fig. 4). Replication of the 2a/L-GDD replicon was increased ∼17-fold in the SEC14L2-expressing cell line at 72 h p.e.; a slightly lower enhancement was observed for the 2a/l-GDD (CpG/UpA-low) construct (Fig. 4A). Replication enhancement was synergistic, with a 340-fold increase in luciferase expression levels for 2a/l-GDD in 23 cells compared to that for 2a/L-GDD in Huh7.5 cells. Consistent with a role for SEC14L2 expression in enhancing replication, luciferase expression levels from the defective 2a/L-GND and 2a/l-GND replicons were comparable between cell lines (Fig. 4A).

A comparable 10-fold enhancement of the replication of the Con1 1b/L-GDD and 1b/l-GDD replicons was observed in 23 cells, with similarly no effect on their replication-defective counterparts (Fig. 4B). SEC14L2 expression and lowering of CpG/UpA frequencies had a synergistic effect, leading to an overall 1,200-fold replication enhancement compared to that of the original replicon in Huh7.5 cells. Replicons based on genotype 3a and 4a backbones (3a/L-GDD and 4a/L-GDD) showed ∼1-log increased luciferase expression. A much larger cell line-dependent increase was observed in the neomycin-deleted 3a replicon 3a/l-GDD (Fig. 3C and D).

### Mechanism of replication enhancement.

**FIG 5:**
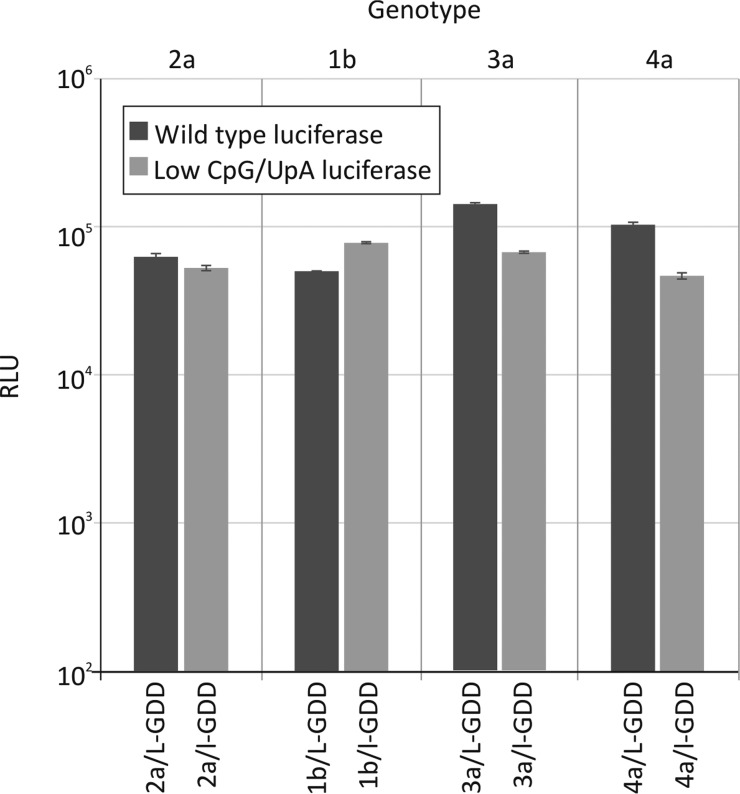
*In vitro* translation assay of wild-type and low-CpG/UpA luciferase replicons. Shown are measurements of luciferase activity of translation products of WT and CpG/UpA-low HCV replicons of genotypes 1 to 4 after a 30-min incubation. Bars show the means of data from two replicates; error bars show standard deviations.

The increased replication of replicons with alterations in dinucleotide composition may have originated from differences in the efficiencies of translation of the luciferase gene through associated alterations in codon usage or codon pair bias (25–27). Alternatively, as demonstrated for echovirus 7, changes in CpG and UpA frequencies may influence the cellular response to infection and induce greater restriction of replication (17). To investigate the effects of CpG and UpA dinucleotide frequency changes on translation, replicons from all four genotypes containing the original (L) or modified (l) luciferase genes were assayed for translation efficiency in an *in vitro* translation assay (Fig. 5). Despite the large differences in codon usage and codon pair bias between the insect-derived luciferase gene and the CpG/UpA-minimized mutant sequence (Table 1), expression levels of the original and mutant forms of the luciferase gene in all four replicons of genotypes 1 to 4 were similar. The two forms of the luciferase gene showed at most 2-fold differences in translation efficiency but with no evidence for any consistently higher expression levels of the CpG/UpA-low luciferase sequences than those of the wild type (Fig. 5). To ensure that the assay mixture was not saturated with RNA that narrowed differences in expression levels, the assay was repeated by using different RNA transcript amounts. Transfection of 4 times more and 4 times less RNA confirmed that the readouts for the assay concentrations used were in the linear range (see Fig. S2 in the supplemental material).

Replicons containing modified luciferase gene sequences showed comparable stabilities posttransfection. In the absence of replication, RNA levels of both genotype 1 and 2a replicons were comparable at 4 h posttransfection (see Fig. S3 in the supplemental material).

**FIG 6:**
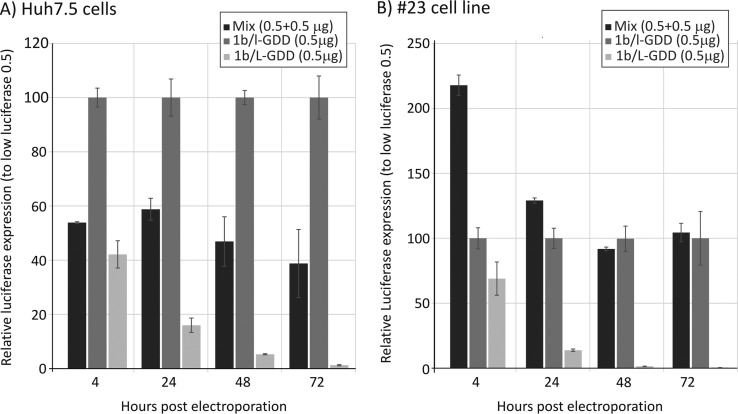
Coelectroporation of CpG/UpA-low and WT luciferase-containing replicons. Shown are luciferase expression levels in Huh7.5 cells (A) and 23 cells (B) after electroporation of CpG/UpA-low, WT, or both replicons; the *y*-axis scale is normalized to luciferase expression by 1b/l-GDD (100%). Error bars depict standard deviations.

As the restriction in replication engendered by increased frequencies of CpG and UpA dinucleotides was not mediated by differences in translation efficiency or greater RNA instability, we next investigated whether the inhibition of replication in replicons expressing native (CpG/UpA-high) luciferase genes was mediated by the replicon containing the gene sequence (in *cis*) or induced a global change in the cell in permissivity to HCV replication (in *trans*). 1b/L-GDD and 1b/l-GDD (containing WT and CpG/UpA-low luciferase gene sequences, respectively) were coelectroporated into Huh7.5 cells (Fig. 6A). The presence of 1b/L-GDD RNA minimally reduced the replication of 1b/l-GDD (∼50%) compared to the expression levels of 1b/l-GDD electroporated alone. The experiment was repeated with the SEC14L2-expressing cell line, where there was no effect on the expression of luciferase compared to that with electroporation of 1b/l-GDD alone (Fig. 6B). These findings provide evidence that the effects of dinucleotide composition are mediated locally (in *cis*) on the RNA molecule possessing the altered dinucleotide composition rather than such a sequence inducing whole cellular restriction on replication (e.g., mediated through the induction of interferon beta).

### Use of enhanced transient-transfection assays to measure susceptibility to DAAs.

**FIG 7:**
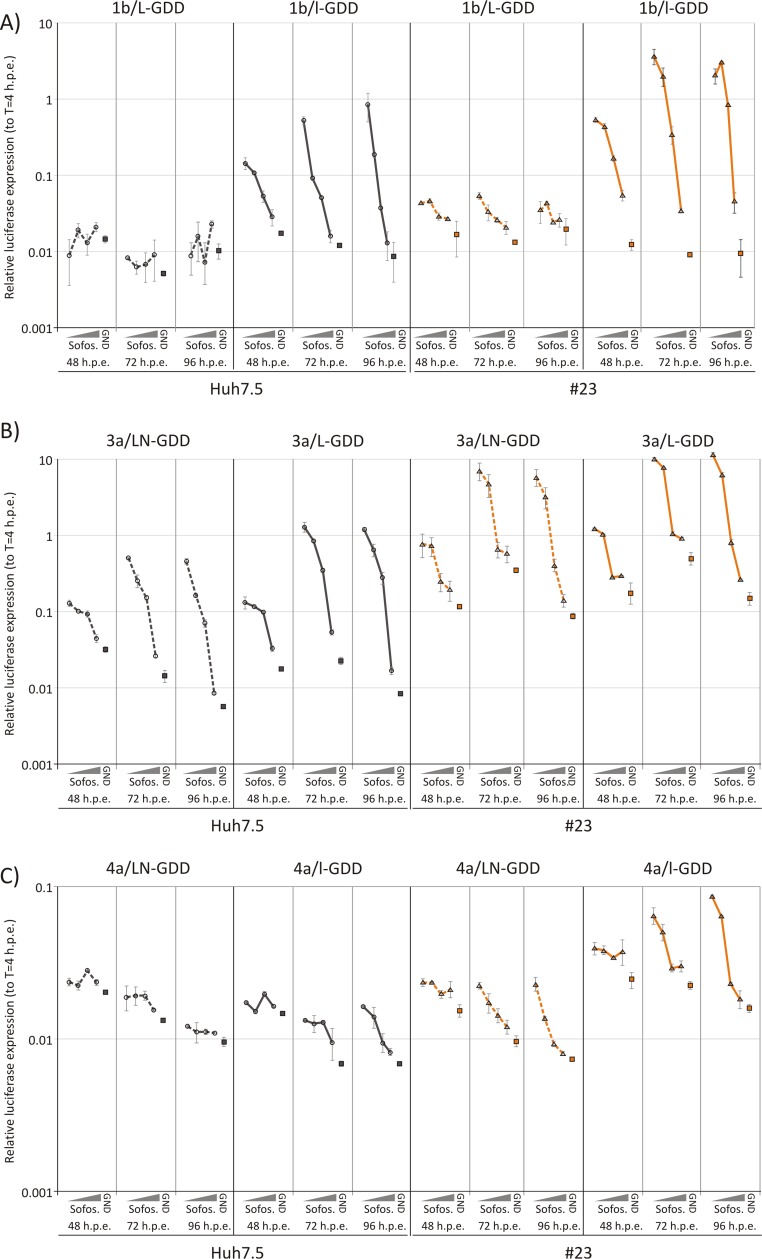
Testing of susceptibility of original and modified replicons to sofosbuvir (Sofos.). Shown are data for the inhibition of replication of CpG-low and WT luciferase replicons 1b/l-GDD and 1b/L-GDD (A), 3a/LN-GDD and 3a/L-GDD (B), and 4a/LN-GDD and 4a/l-GDD (C) with differing concentrations of sofosbuvir. The experiment was performed with the Huh7.5 and 23 cell lines. Error bars depict standard deviations.

For testing the inhibitory capacity of HCV antiviral agents in transient-replication assays, achieving sufficient replication levels to quantify degrees of inhibition is essential. We therefore investigated whether the enhanced replication achieved by replacing the wild-type luciferase or culture in an SEC14L2-expressing cell line facilitated the evaluation of the NS5B inhibitor sofosbuvir in genotypes 1b, 3a, and 4a. Cells were electroporated with each replicon in the presence of a range of sofosbuvir concentrations spanning the previously established 50% inhibitory concentrations (IC_50_s) (28, 29). Luciferase expression levels were measured at 48, 72, and 96 h postelectroporation. Levels of nonreplicating replicons were included as baseline levels of luciferase expression. The 1b/L-GDD and 4a/L-GDD replicons replicated at such low levels in the Huh7.5 cell lines that replication inhibition by sofosbuvir could not be detected (Fig. 7, left two panels). However, performing the assay with SEC14L2-expressing cells improved replication and enabled a concentration-dependent inhibition of replication by sofosbuvir to be detected (Fig. 7A and C, columns 7 to 9). A major enhancement was observed by using the 1b/l-GDD replicon in Huh7.5 cells, which was improved even more when SEC14L2-expressing cells were used. This same pattern, but to a lesser extent, was observed for genotype 4a. Although the wild-type genotype 3a replicon already showed usable titration data with Huh7.5 cells, the removal of neomycin and especially the use of SEC14L2-expressing cells improved overall replication and enabled a robust estimation of IC_50_s for sofosbuvir for this genotype (Table 2).

**TABLE 2:** IC_50_s for sofosbuvir in genotypes 1b (Con1), 3a (S52), and 4a (ED43)

Cell line	Replicon	Mean IC_50_ ± SD^*a*^		
		48 h p.e.	72 h p.e.	96 h p.e.
Huh7.5	1b/L-GDD	—	—	—
	1b/l-GDD	—	—	—
	3a/LN-GDD	48 ± 4	42 ± 1	57 ± 2
	3a/L-GDD	53 ± 8	47 ± 9	40 ± 2
	4a/LN-GDD	—	—	—
	4a/l-GDD	—	—	49 ± 27
23	1b/L-GDD	—	—	—
	1b/l-GDD	95 ± 8	42 ± 4	55 ± 6
	3a/LN-GDD	35 ± 3	38 ± 6	29 ± 1
	3a/L-GDD	42 ± 3	41 ± 2	29 ± 1
	4a/LN-GDD	—	—	28 ± 10
	4a/l-GDD	—	45 ± 15	39 ± 1

a IC_50_s are the concentrations of sofosbuvir that inhibit the HCV replicon by 50%. —, not determined (insufficient replication).

## DISCUSSION

Despite reproducing only the intracellular replication steps of the HCV life cycle, replicons are a valuable tool in HCV research, especially in drug discovery programs, and have been instrumental in the discovery of the first DAAs (30), including sofosbuvir (31), simeprevir (32, 33), and boceprevir (34). In addition to susceptibility testing, *in vitro* systems play an essential role in monitoring the phenotypic effects of resistance-associated mutations that arise during DAA treatment on drug susceptibility (35, 36). Resistance mutations are frequently genotype specific, and for their effects to be quantified, inhibition assays require that these mutations be tested in the same genotypic background *in vitro*. DAA susceptibility and resistance testing should also be performed without the confounding effect of unpredictable cell culture-adaptive mutations that are likely to arise with antibiotic selection.

To date, however, most information on susceptibility and resistance testing has been based on *in vitro* assays using cell lines stably expressing HCV replicon RNA, typically H77 (genotype 1a) and Con1 (genotype 1b) (36). Selection of stable cell lines prior to testing is also normally used in the analysis or selection of resistance mutations (37–39). However, construction of stably transformed cell lines is a time-consuming procedure and is also likely to introduce additional cell culture-adaptive mutations that may also influence DAA susceptibility, complicating comparisons with wild-type replicons. Therefore, the use of a transient-expression assay with a range of genotypes would be highly advantageous in terms of speed, simplicity, and avoidance of cell culture-induced artifacts. Although some of these assays have been used in the analysis of mutations associated with resistance to sofosbuvir (29), the low level of replication generally achieved means that such studies are problematic to extend to a wider range of HCV strains and genotypes.

To improve the replication of replicons in transient-expression assays, we first replaced the wild-type luciferase gene with a modified reporter gene. In this sequence, all CpG dinucleotides and as many UpA dinucleotides as possible were removed while retaining the same coding sequence and maintaining comparable codon usage. In both genotypes 1b (Con1) and 2a (JFH1), this replacement resulted in a 2-log improvement in replication rates compared to those of replicons containing the original wild-type luciferase sequence (Fig. 1A and B). This enhancement of replication was comparable to that exhibited for echovirus 7 replicon with a similar replacement of the luciferase gene (17). How this enhancement of both initial gene expression and subsequent increased replication is mediated remains uncertain, although observations in this study can rule out some mechanisms. First, the absence of any consistent difference in the translation efficiencies of original and modified luciferase genes in different replicon constructs clearly demonstrates that the enhancement of replication of CpG/UpA-low replicons was not mediated by a translational mechanism. These findings are consistent with previous studies demonstrating comparable translation efficiencies of mutants of E7 with regions of the genome with altered dinucleotide frequencies and codon pair bias (18). Similarly, mutants of poliovirus differing in codon pair frequencies/dinucleotide frequencies showed relatively small differences in translation rates that were not predictive of their replicative ability (26). A detailed investigation of the effects of a range of compositional variables on the replication of poliovirus reported that CpG and UpA dinucleotide frequencies primarily influenced the replication of poliovirus and were unaffected by variation in codon usage, codon pair bias, and other metrics predictive of translational optimization, such as the CAI (27). Modification of codon pair bias and CpG/UpA dinucleotide frequencies in echovirus 7 similarly indicated the primary influence of dinucleotide frequencies on virus replication (18). In a broader context, differences in the expression of the luciferase gene mediated purely through translational effects cannot contribute to the replication fitness of HCV replicons, as its purpose is simply to act as a reporter gene. The enhancement of replication of replicons containing CpG/UpA-low luciferase coding sequences must therefore be mediated through alternative mechanisms.

The minimal or absent interference with the replication of 1b/l-GDD by the wild-type 1b/L-GDD replicon additionally argues against global restriction in permissivity for viral replication that would be expected in interferon-primed cells. The lack of interference between replicons is, however, compatible with the hypothesis advanced previously to explain the restriction of CpG/UpA-high mutants of E7. In that report, possession of high-CpG and -UpA dinucleotides appeared to induce a localized stress response in the cell that influenced the ability of viruses to establish replication complexes (17). The HCV replicon system established in this study provides a valuable tool for future dissection of the restriction mechanisms associated with altered dinucleotide frequencies.

The clear replication enhancement observed in the genotype 1b and 2a replicons containing the CpG/UpA-low luciferase sequence was not fully reproduced in genotypes 3a and 4a but showed a more subtle and complicated pattern. The original versions of both replicons expressed luciferase as a fusion protein with neomycin (21). The removal of neomycin in genotype 3a increased replication, although in contrast to other replicons, the replacement of the luciferase component of the fusion protein with the CpG/UpA-low sequence negatively impacted the replication of the genotype 3a replicon (Fig. 2C) for reasons that remain undetermined. In the genotype 4a replicon, removal of the neomycin gene similarly resulted in enhanced replication at late time points, but in this case, the introduction of the low-CpG/UpA luciferase further increased replication to a degree similar to that observed for genotypes 1b and 2a. Understanding how the restrictions in replication mediated through dinucleotide composition interact with the limited or complete inability of many genotypes or strains of HCV to replicate in cell culture will require a much better understanding of the cellular pathways that mediate these replication phenotypes and their potential for interaction.

As a further manifestation of the complexity of the restriction of HCV replication *in vitro*, substantial increases in the rates of replication of replicons with all four genotype backgrounds were achieved in cells overexpressing SEC14L2. SEC14L2 was originally reported to enhance the replication of non-cell-culture-adapted isolates of HCV that are not resistant to lipid peroxidation (19). We investigated whether the same positive effect on replication could be achieved for replicons in a transient-expression assay format. Transduction of parental Huh7.5 cells and consequent selection yielded colonies with various levels of SEC14L2 mRNA and protein expression (Fig. 3A). Knockdown of SEC14L2 by siRNA was used to confirm the involvement of this gene and that it was not an artifact of transduction or selection (Fig. 2C and D). Transient-expression assays with HCV replicons showed a broadly consistent 10-fold increase in replication in cells expressing high levels of SEC14L2 compared to that in the parental Huh7.5 cell line (Fig. 4A to C). The expression of SEC14L2 had no effect on the expression of luciferase in replication-incompetent (GND or GAA) replicons, suggesting that SEC14L2 directly influenced replication rather than RNA stability and/or translation. However, in contrast to previously reported results (19), we observed a consistent and substantial increase in the rate of replication in the lipid peroxidation-resistant genotype 2a JFH1 replicon, whose replication was not enhanced in stably transfected cell lines. Differences in assay systems may have contributed to this difference. First, our experiments were performed with a cell line with SEC14L2 expressed from a transgene that is not under the same regulatory control as the native gene. Second, we electroporated cells instead of using transfection; this may deliver RNA at a different location and efficiency and influence the efficiency of initial translation and gene expression and the consequent replication efficiency.

Irrespective of the likely complex mechanisms underlying the restriction of HCV replication in cell culture, this study achieved its original goal of improving the replication of replicons, which enables their use in transient-expression assays. This enabled a pilot study of the susceptibilities of different genotypes to the NS5B inhibitor sofosbuvir (Fig. 7). The enhancement of replication in SEC14L2-exressing cells and the change to the low-CpG/UpA luciferase reporter gene enabled robust measurement of IC_50_s for genotypes 1 to 4 (Table 2), generating values that were comparable to those reported previously for other assay systems (28, 29).

In summary, we have shown that reducing the number of CpG and UpA dinucleotides in HCV subgenomic replicons can greatly enhance replication levels but with some variability between genotypes. Combined with further increases in HCV replication in cell expressing SEC14L2, the 30- to 1,000-fold increases in replication achieved across all four genotypes will greatly facilitate susceptibility and resistance mutation testing in a convenient and rapid transient-expression assay format.

## Supplementary Material

Supplemental material
