# Supplementary material for: Enhancement of the Replication of Hepatitis C Virus Replicons of Genotypes 1 to 4 by Manipulation of CpG and UpA Dinucleotide Frequencies and Use of Cell Lines Expressing SECL14L2 for Antiviral Resistance Testing
Source: Antimicrob Agents Chemother. 2016 Apr 22;60(5):2981–92. doi: 10.1128/AAC.02932-15 (PMC4862521; doi:10.1128/AAC.02932-15)
Supplement: Supplemental material [file supp_60_5_2981__index.html]

Supplemental material 

# Enhancement of the Replication of Hepatitis C Virus Replicons of Genotypes 1 to 4 by Manipulation of CpG and UpA Dinucleotide Frequencies and Use of Cell Lines Expressing SECL14L2 for Antiviral Resistance Testing

## Supplemental material

- Supplemental file 1 -

  Fig. S1-S3

  PDF, 557K
