# Supplementary material for: Enhancement of the Replication of Hepatitis C Virus Replicons of Genotypes 1 to 4 by Manipulation of CpG and UpA Dinucleotide Frequencies and Use of Cell Lines Expressing SECL14L2 for Antiviral Resistance Testing
Source: Antimicrob Agents Chemother. 2016 Apr 22;60(5):2981–92. doi: 10.1128/AAC.02932-15 (PMC4862521; doi:10.1128/AAC.02932-15)
Supplement: Supplemental material [file AAC.02932-15_zac005165151so1.pdf]

Supplementary Data

Sequence of the CpG/UpA-minimised luciferase gene (l):

>Luc_CGUAL
GGCGCGCCATGGAAGATGCCAAAAACATCAAGAAAGGCCCAGCCCCATTCTATCCTCTGG
AGGATGGAACAGCTGGAGAGCAACTGCACAAGGCCATGAAGAGATATGCCCTGGTTCCTG
GAACAATTGCTTTCACAGATGCACACATAGAGGTGAACATCACATATGCTGAATACTTTG
AAATGTCTGTCAGGTTGGCAGAAGCCATGAAAAGATATGGGCTGAACACAAATCACAGAA
TTGTTGTGTGCAGTGAAAACTCTCTTCAATTCTTCATGCCTGTGTTGGGGGCTTTGTTCA
TTGGAGTTGCAGTTGCTCCTGCTAATGACATTTACAATGAAAGAGAATTGCTCAACAGCA
TGAACATTTCCCAGCCCACAGTGGTGTTTGTTTCCAAAAAGGGGTTGCAAAAAATTTTGA
ATGTGCAAAAAAAATTGCCAATCATCCAGAAAATCATCATCATGGATTCCAAAACTGATT
ACCAGGGATTTCAGTCCATGTACACATTTGTCACATCTCATCTGCCTCCTGGTTTCAATG
AATATGATTTTGTGCCAGAGTCCTTTGACAGAGACAAAACAATTGCACTGATCATGAACT
CCTCTGGATCCACTGGGTTGCCCAAGGGTGTGGCCCTTCCCCACAGAACTGCCTGTGTCA
GATTCTCACATGCCAGAGATCCCATTTTTGGCAATCAAATCATTCCAGACACTGCCATTT
TGAGTGTTGTTCCATTCCATCATGGTTTTGGAATGTTCACCACACTTGGATATTTGATCT
GTGGATTCAGAGTTGTCTTGATGTACAGATTTGAAGAAGAGCTGTTTTTGAGATCCCTTC
AGGATTACAAAATTCAAAGTGCCTTGCTGGTTCCAACCCTTTTTTCATTCTTTGCCAAAA
GCACTCTGATTGACAAATATGATTTGTCAAATTTGCATGAAATTGCTTCTGGGGGGGCAC
CTCTTTCAAAAGAAGTGGGGGAAGCAGTTGCAAAAAGATTCCATCTTCCAGGGATCAGAC
AAGGATATGGGCTCACTGAGACAACATCAGCCATTCTGATCACACCTGAGGGGGATGACA
AACCAGGTGCAGTGGGGAAAGTTGTTCCATTTTTTGAAGCCAAGGTTGTGGATCTGGACA
CTGGGAAAACTCTGGGGGTGAATCAGAGAGGGGAATTGTGTGTCAGAGGACCCATGATCA
TGTCAGGTTATGTCAACAATCCAGAAGCAACCAATGCCTTGATTGACAAGGATGGATGGC
TGCATTCTGGAGACATTGCTTACTGGGATGAAGATGAACACTTCTTCATTGTTGACAGAT
TGAAGTCTTTGATCAAATACAAAGGATACCAGGTGGCCCCTGCTGAATTGGAGTCCATTT
TGTTGCAACACCCCAACATCTTTGATGCAGGGGTGGCAGGTCTTCCTGATGATGATGCTG
GTGAACTTCCAGCTGCAGTTGTTGTTTTGGAGCATGGAAAGACAATGACTGAAAAAGAGA
TAGTGGATTATGTTGCCAGTCAAGTGACAACTGCCAAAAAGTTGAGGGGAGGAGTTGTGT
TTGTGGATGAAGTTCCAAAAGGTCTGACTGGAAAACTGGATGCAAGAAAAATCAGAGAGA
TCCTCATCAAGGCCAAGAAGGGGGGAAAGTCCAAATTGTAAGTTTAAAC

Unique restriction sites at the 5‘ end (*Asc*I) and 3’ end (*Pme*I) underlined.

Fig. S1

Expression of luciferase at early time points post-transfection from Replication competent and defective genotype 2a replicons

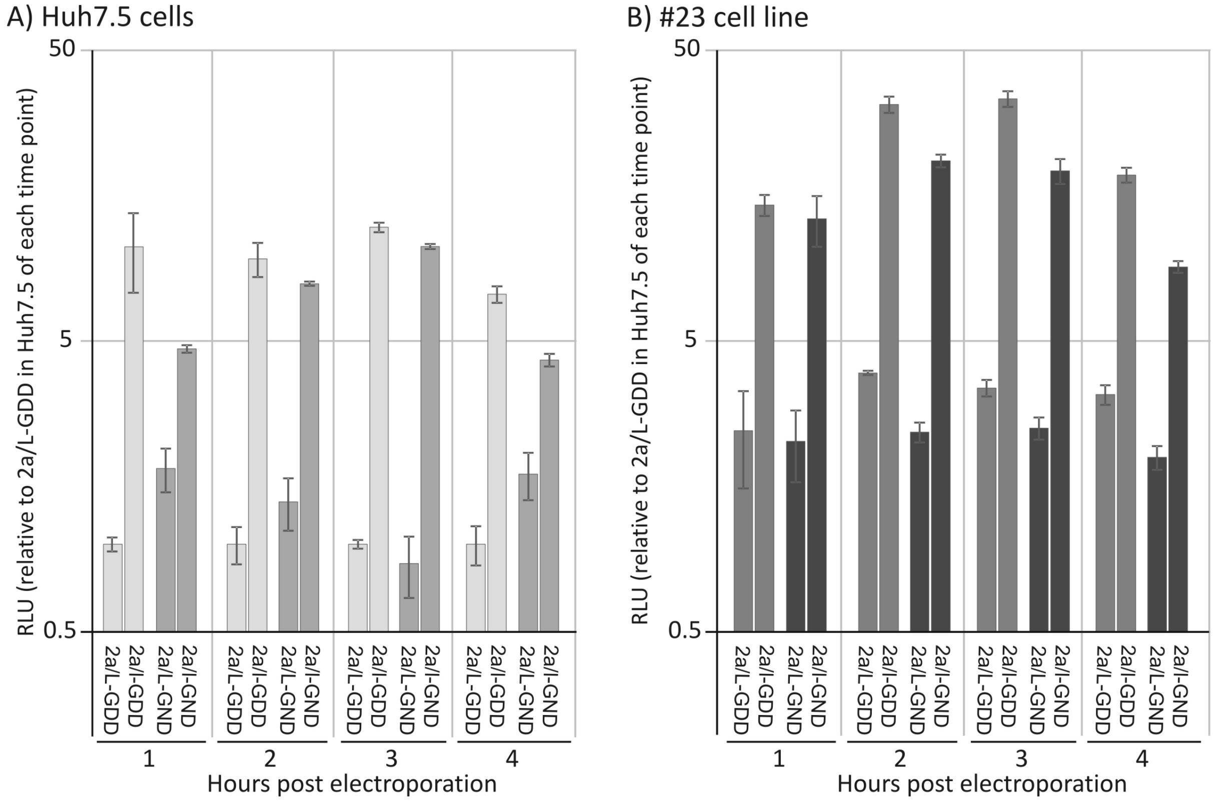

Luciferase expression in (A) Huh7.5 cells and (B) #23 cells after electroporation of genotype 2a replicons with WT and CpG/UpA-minimised luciferase gene sequences; y-axis scale is normalised to luciferase expression by 2a/L-GDD. Error bars depict SEMs.

Fig. S2

Effect of replicon RNA amount on luciferase detection

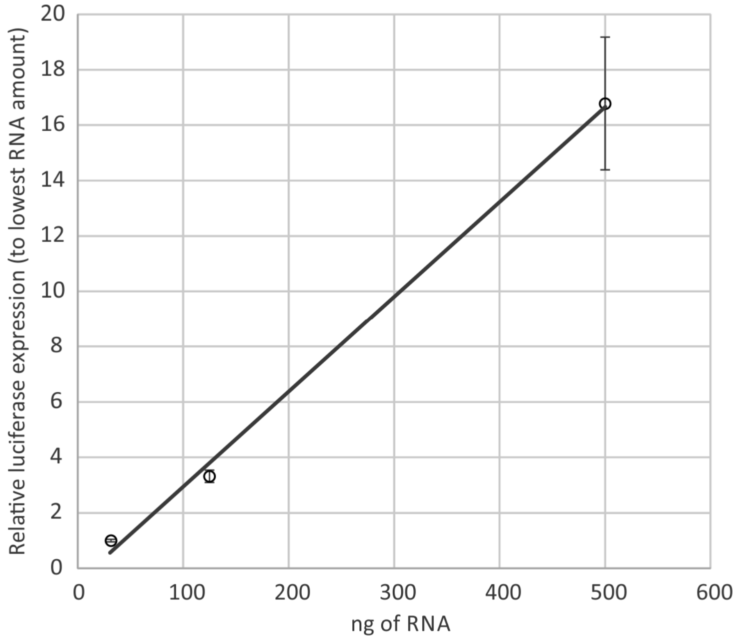

Translation of 1b/L-GND using 4 times less and 4 times more than the amount used in the comparison of different replicons (Figure 6) in a rabbit reticulocyte assay. Luciferase expression was measured after a 30 minute incubation time. Shown are the average of two replicates for each replicon; error bars show standard deviations.

FIGURE S3

Stability of HCV replicon RNA post transfection

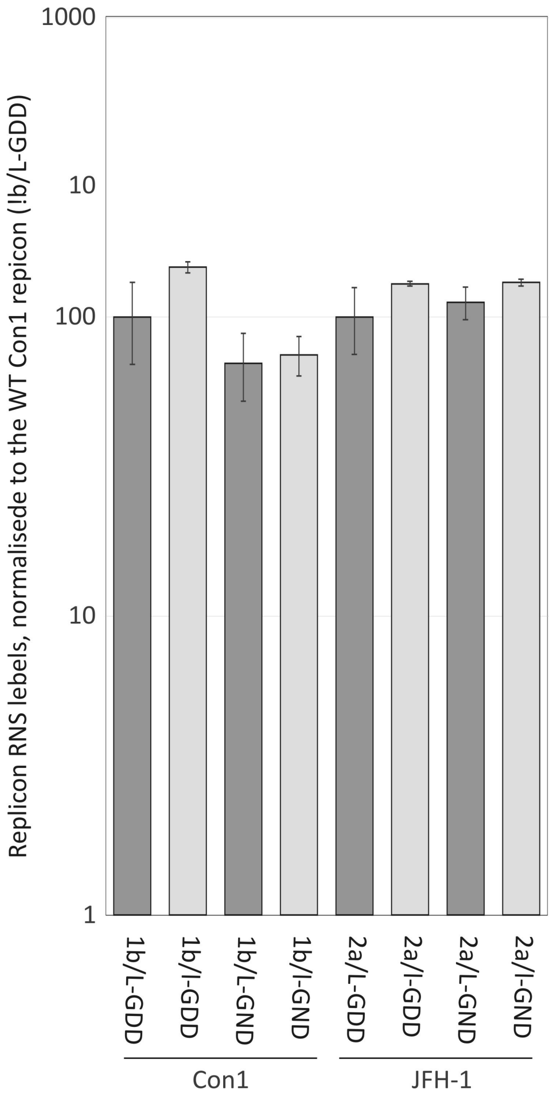

Quantitative PCR for HCV RNA at hours post-transfection for genotype 1b (Con1) and 2a (JFH-1) replicons. Values have neen normalised to the RNA level of the Con1 WT replicon (1.0; y-axis). Rrror bars show standard deviations
